# Two Fluorescent Probes for Recognition of Acetylcholinesterase: Design, Synthesis, and Comparative Evaluation

**DOI:** 10.3390/molecules29091961

**Published:** 2024-04-25

**Authors:** Xia Lin, Qingyuan Yi, Binyang Qing, Weisen Lan, Fangcheng Jiang, Zefeng Lai, Jijun Huang, Qing Liu, Jimin Jiang, Mian Wang, Lianjia Zou, Xinbi Huang, Jianyi Wang

**Affiliations:** 1 Guangxi Key Laboratory of Special Biomedicine, Medical College, Guangxi University, Nanning 530004, China; linxia2873@163.com (X.L.); 2028391028@st.gxu.edu.cn (Q.Y.); 18278019102@163.com (W.L.); 2 Faculty of Pharmacy, Guangxi Health Science College, Nanning 530023, China; lianjiazou_gxmc@sina.com; 3 School of Chemistry and Chemical Engineering, Guangxi University, Nanning 530004, China; 4 College of Life Science and Technology, Guangxi University, Nanning 530004, China; lycansaz@gmail.com (B.Q.); mianwang@gxu.edu.cn (M.W.); 5 Pharmaceutical College, Guangxi Medical University, Nanning 530021, China; fangchengjiang@sr.gxmu.edu.cn (F.J.); laizefeng@gxmu.edu.cn (Z.L.); 6 Guangxi Zhuang Autonomous Region Drug Administration, Nanning 530029, China; jijunhuang_gxdrug@163.com (J.H.); qingliu_gxdrug@163.com (Q.L.); jiminjiang_gxdrug@163.com (J.J.)

**Keywords:** acetylcholinesterase, probe, comparative evaluation, lung carcinoma cell, apoptosis, visualized monitoring

## Abstract

In this study, two “on–off” probes (BF_2_-cur-Ben and BF_2_-cur-But) recognizing acetylcholinesterase (AChE) were designed and synthesized. The obtained probes can achieve recognition of AChE with good selectivity and pH-independence with a linear range of 0.5~7 U/mL and 0.5~25 U/mL respectively. BF_2_-cur-Ben has a lower limit of detection (LOD) (0.031 U/mL), higher enzyme affinity (K_m_ = 16 ± 1.6 μM), and higher inhibitor sensitivity. A responsive mechanism of the probes for AChE was proposed based on HPLC and mass spectra (MS) experiments, as well as calculations. In molecular simulation, BF_2_-cur-Ben forms more hydrogen bonds (seven, while BF_2_-cur-But has only four) and thus has a more stable enzyme affinity, which is mirrored by the results of the comparison of K_m_ values. These two probes could enable recognition of intracellular AChE and probe BF_2_-cur-Ben has superior cell membrane penetration due to its higher log *p* value. These probes can monitor the overexpression of AChE during apoptosis of lung cancer cells. The ability of BF_2_-cur-Ben to monitor AChE in vivo was confirmed by a zebrafish experiment.

## 1. Introduction

Acetylcholinesterase (AChE) is a component of the cholinergic system that terminates the conduction of nerve impulses in cholinergic neurons by hydrolysing the neurotransmitter acetylcholine. AChE is not only abundant in the cerebral cortex and cerebellum, but also widely distributed in a variety of non-excitatory tissues in the human body except the nervous system, such as liver, lung, kidney, colon, etc. [1]. It is involved in a wide range of cellular processes, including cell development and maturation [2,3]. The induction of apoptosis is usually accompanied by an increase of AChE expression. Conversely, AChE deficiency could protect normal cells by reducing apoptosis, which has been demonstrated in mouse models [4,5,6,7,8,9]. AChE expression in a variety of cancerous tissues is usually lower than that in paraneoplastic tissues, and it has a growth inhibitory effect on cancer cells [10,11,12]. Therefore, detection of the content and activity of AChE in apoptosis is of great significance for precision diagnosis of related diseases.

The common methods to detect AChE involve colorimetric methods [13], chemiluminescent methods [14], and electrochemical methods [15,16]. However, the disadvantages of low detection sensitivity, expensive equipment, and non-real-time monitoring limit the wide application of these methods. Fluorescence technology has recently attracted much attention from academic and industrial communities because of its high sensitivity, high specificity, noninvasiveness, deep tissue penetration, and real-time detectability in vivo and in vitro. The existing fluorescent probes to recognize AChE can be mainly categorized into two types: The first one is an indirect type. AChE can decompose the substrate acetylcholine thioate (ATCh) into acetic acid and thiocholine (TCh). The sulfhydryl group (-SH) on TCh with reducing, complexing and strong nucleophile properties, can further react with luminescent substances (fluorescent proteins [17], quantum dots [18,19], metal nanoclusters [20,21], conjugated polymers [22], organic fluorescent dyes [23,24,25], etc.) to produce detectable signals. For such probes, the choice of luminescent material largely determines the biocompatibility and preparation cost. For example, quantum dots are toxic and metal nanoclusters are poorly biocompatible with cell membranes, so probes using them as a source of fluorescent signals can rarely be applied to intracellular AChE monitoring. Although fluorescent proteins have good biocompatibility, their sources are expensive (usually obtained through genetic engineering). 

Fluorescent dyes can effectively circumvent the above-mentioned problems. For example, the indirect-type probe HBTP can recognize Cu^2+^ and AChE successively and has been applied to human embryonic kidney cells HEK293 [24], and the probe NFL-SF can recognize AChE and has been applied to human cervical cancer cells (HeLa) and human breast cancer cells (MCF-7) [25]. 

For the direct type, AChE acts directly on the ester or amide bond of the probe and breaks its chemical bond, changing its conjugation system and generating a change in the fluorescence signal. For this type of probe, phorbol ester, hemiphorbol ester, boron dipyrrole fluoride (BODIPY) and tricyanofuran (TCF) are usually used as fluorescent groups, and N,N-dimethylformamide ester, acetylcholine ester, and huprine amide are selected as recognition groups, realizing recognition of AChE in cells and muscle nerve tissue [26,27,28,29,30,31,32]. However, the LODs of these direct-type probes are a little high, some of the fluorophores are expensive, some of the preparation methods are complex, and the yield is low. Furthermore, these probes focus mostly on AChE in brain cells or tissues and rarely monitor AChE in lung cancer cells.

**Figure 1:**
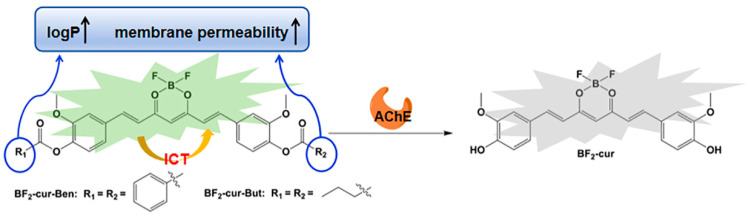
Design principle of the aimed probes.

Curcumin is an inexpensive substance derived from the natural plant turmeric and can promote apoptosis in a variety of cancer cells, including lung cancer cells [33,34,35,36]. But its physiological activities are affected by its structural instability. The structure of curcumin can be stabilized by forming a hexatomic ring with BF_2_ at the 3, 5-position. Excitingly, the cyclized structure of curcumin exhibits significant anti-proliferative effects on leukemia cells, colorectal cancer cells, and lung cancer cells [37,38,39,40,41], as well as excellent fluorescence properties. To obtain ideal probes with favourable oil-water distribution coefficients (favourable membrane permeability), in this study, two intramolecular charge transfer (ICT) based probes BF_2_-cur-Ben and BF_2_-cur-But were designed with BF_2_-cur as the fluorophore and benzoyl ester and butyryl ester as the recognition groups (Figure 1), which is expected to enable the visualization and monitoring of overexpressed AChE during apoptosis in lung cancer.

## 2. Results and Discussion

### 2.1. Response and Detection of the Probes for AChE In Vitro

We first tested the fluorescence emission of these two probes in different polar solvents. As shown in Figure S1, although there are some differences in the fluorescence emission wavelengths versus the emission intensities in different solvents, in general, these differences are within an acceptable range to avoid potential artifacts. Considering that the target AChE is a biological enzyme that requires specific buffer conditions to remain active, we finally chose the solvent system DMSO/PBS = 5/95 (*v*/*v*). In addition, the Lambert–Beer plot (Figure S2) of the two probes indicated that the solubility of the probes in this solvent system is relatively good. See from Figure S3, the probe BF_2_-cur-Ben and BF_2_-cur-But solution exhibited UV absorption peaks at 445 nm and 476 nm, respectively. For BF_2_-cur-Ben, once AChE was added, these two UV absorption peaks weakened, and a new strong absorption peak was found at 411 nm, which had undergone a significant blue shift. Then, 476 nm and 411 nm were selected as excitation wavelengths, respectively, and the fluorescence change amount was much larger in the former (8.4 times more in the former than in the latter). The spectral profiles for BF_2_-cur-But were similar, but with the same probe and AChE concentration, the fluorescence changes were even more dramatic for BF_2_-cur-Ben.

**Responsive time**. To explore the response speed of the probes for AChE, we conducted responsive experiments at different times. See in Figure S4, for BF_2_-cur-Ben, the decreasing trend (slope) was maintained from 10 min to 120 min. Considering the efficiency and fluorescence effect, 60 min was finally selected for the subsequent experiments. For BF_2_-cur-But, 20 min was enough. Obviously, the two probes can achieve an evident response within 60 min, which meets the requirements for a rapid response in biological monitoring.

**Figure 2:**
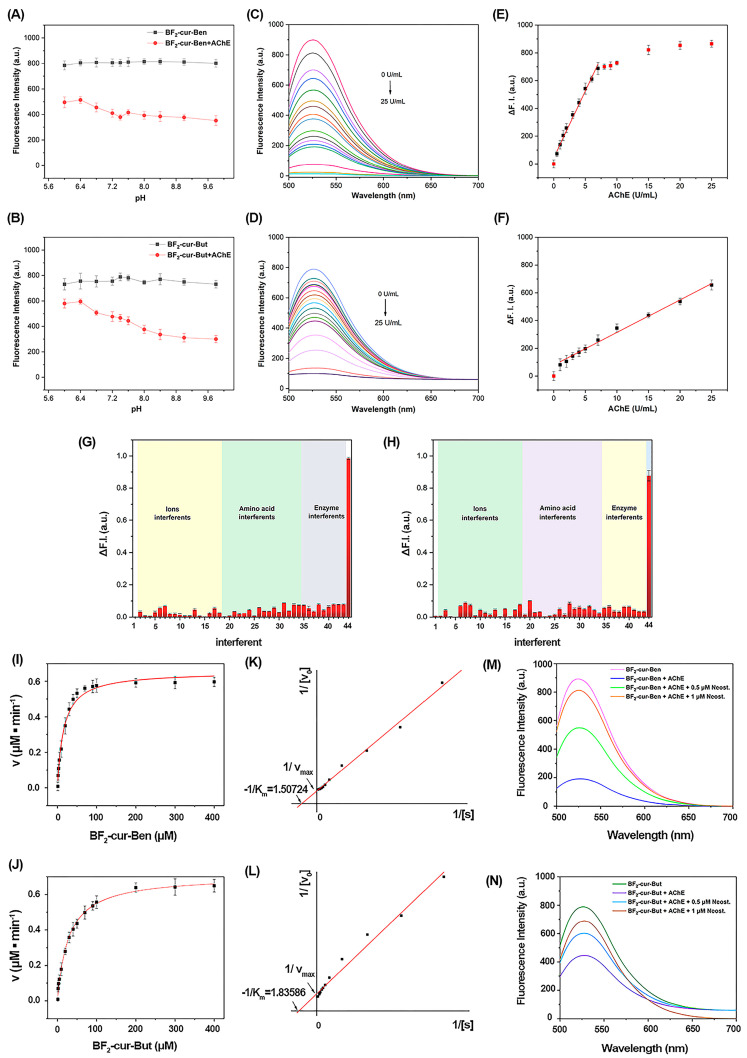
pH adaptation (**A**,**B**), fluorescence titration (**C**,**D**) and standard curve (**E**,**F**) of BF_2_-cur-Ben and BF_2_-cur-But to AChE, respectively. Fluorescence response of BF_2_-cur-Ben (**G**) and BF_2_-cur-But (**H**) with different interferences. 1. blank, 2. K^+^, 3. Na^+^, 4. Ca^2+^, 5. Fe^3+^, 6. Fe^2+^, 7. Mg^2+^, 8. Zn^2+^, 9. Cu^2+^, 10. Ba^2+^, 11. NH_4_^+^, 12. CO_3_^2−^, 13. Br^−^, 14. C_2_O_4_^2−^, 15. CH_3_COO^−^, 16. ClO^−^, 17. HS^−^, 18. Lys, 19. Pro, 20. Met, 21. Gln, 22. Thr, 23. Ala, 24. Phe, 25. Ile, 26. Gly, 27. Leu, 28. Glu, 29. Arg, 30. His, 31. Tyr, 32. Vc, 33. GSH, 34. Cys, 35. CEs, 36. BChE, 37. α-chymotrypsin, 38. Lysozyme, 39. Phospholipase, 40. Glucose Oxidase, 41. Elastase, 42. Trypsin, 43. BSA, 44. AChE. Michaelis-Menten curve (**I**,**J**) and Lineweaver–Burk curve (**K**,**L**) of BF_2_-cur-Ben and BF_2_-cur-But catalysed by AChE. Effect of different concentrations of inhibitor neostigmine on fluorescence intensity (**M**,**N**). Error bars are ± SD (*n* = 3).

**Effect of pH value on the probes**. As we well known, high or low pH values would damage the structure and function of the cell membrane, resulting in abnormal cell division and proliferation, so herein we evaluated the effect of different pH environments on the probes. As shown in Figure 2A,B, the fluorescence signal of the two probes before and after response to AChE was stable within the pH range of 6.0~9.8. Under physiological conditions of the human body (pH 7.4), the difference value of fluorescence intensity is apparent. Obviously, these two probes are not affected by pH changes, especially under physiological conditions, and show feasibility to detect enzymes in cells.

**Sensitivity**. As shown in Figure 2C–F, the difference value of fluorescence intensity (∆F. I.) of the probe solution increased continuously with the increase of AChE concentration, and ∆F. I. tended to flatten out when the concentration of AChE was greater than 7 U/mL for BF_2_-cur-Ben, but it increased continuously, even up to 25 U/mL for BF_2_-cur-But. BF_2_-cur-Ben showed good linearity (R^2^ = 0.9934) for AChE in the range of 0.5~7 U/mL, and the regression equation was I = 44.0747 + 96.2710 × [AChE], with the standard deviation σ = 3.007, and the LOD was calculated to be 0.031 U/mL. The regression equation and LOD for BF_2_-cur-But were I = 80.1836 + 23.4562 × [AChE] (R^2^ = 0.9939) (in the range of 0.5~25 U/mL) and 0.087 U/mL. Although BF_2_-cur-But had a wider linear range, it also had a higher LOD. Nevertheless, these two probes both possess good sensitivity toward AChE, showing potential in detection of AChE.

**Selectivity**. As shown in Figure 2G,H, the Y-axis represents ∆F. I., and both BF_2_-cur-Ben and BF_2_-cur-But exhibited the most pronounced fluorescence difference toward AChE, despite the concentrations of the other analytes being higher than AChE. In the presence of various amino acids and other interferences (such as BSA, anions, and cations) commonly found in biological organisms, the probes only showed a slight response. Especially for several common biological enzymes, which operate at 10-fold the working concentration of AChE, just like carboxylesterase (CEs) and butyrylcholinesterase (BChE) (homologous to AChE), the fluorescence response value was 7.4%, 5.5% (BF_2_-cur-Ben) and 6.4%, 7.5% (BF_2_-cur-But) that of AChE, respectively. Although BF_2_-cur-Ben is slightly responsive to interfering enzymes and BF_2_-cur-But is slightly responsive to interfering ions, these substances did not induce a significant fluorescence effect at concentrations well above normal physiological levels. 

AChE belongs to the serine peptidases, a family of proteases with Ser as the active centre. Differences in the binding sites of Ser and substrates determine the substrate specificity of homologous enzymes. To further explore the specificity of AChE and BChE for the two probes, molecular docking simulation calculations were performed, and the results are shown in Figure S5 and Table S1. Both BF_2_-cur-Ben and BF_2_-cur-But can form more hydrogen bonds in AChE than in BChE, and the shortest attack distance of Ser is equal or shorter in AChE. Since more hydrogen bonds can enhance the affinity between the substrate and the enzyme [42], both of these probes have a stronger affinity for AChE than BChE; hence can exclude the interference of BChE. Obviously, these two aimed probes present highly specific and good selectivity toward AChE and will not be disturbed by the enzymes, amino acids and ions mentioned above.

**Enzyme kinetics.** As shown in Figure 2I–L, the rate of AChE-catalysed hydrolysis of the probes (BF_2_-cur-Ben and BF_2_-cur-But) was plotted with the concentration of the probes as the horizontal coordinate when the amount of AChE was fixed. The K_m_ value and maximum reaction rate (V_max_) were calculated to be 16 ± 1.6 μM and 0.65 ± 0.018 μM/min for BF_2_-cur-Ben, and 29 ± 2.3 μM and 0.71 ± 0.019 μM/min for BF_2_-cur-But. Comparatively, the K_m_ values of several recently reported probes for identifying AChE range from 4.87 to 141 μM [26,27,29,30,32]. In view of the smaller the Km value, the greater the enzyme affinity, clearly, these two probes have relatively superior affinity for AChE and BF_2_-cur-Ben is better. 

To track the inhibitory effect of inhibitors, we co-incubated the probes with AChE, and different concentrations (0.5, 1 μM) of neostigmine (a typical inhibitor of AChE). Seen from Figure 2M,N, the presence of neostigmine inhibited the fluorescence change, and the inhibition effect became stronger as the concentration of neostigmine increased. The inhibitory effect of BF_2_-cur-Ben was more pronounced for the same concentration of inhibitor. The above results indicate that the fluorescence change is caused by the specific hydrolysis of AChE with the probe, which can be used to indicate the enzyme activity. In comparison, BF_2_-cur-Ben was more sensitive to both the enzyme and inhibitor.

### 2.2. Recognition and Responsive Mechanisms

**Figure 3:**
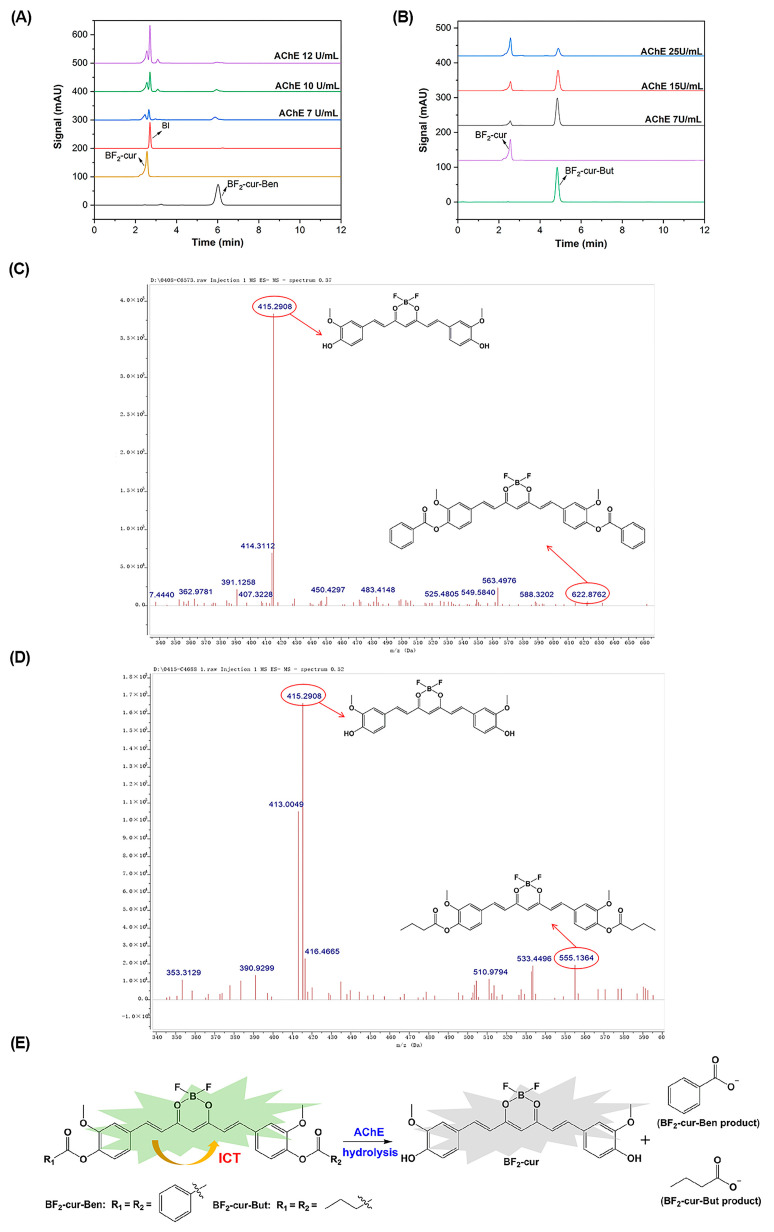
Recognition mechanism of the two probes with AChE. HPLC monitoring of the reaction process of BF_2_-cur-Ben (**A**) and BF_2_-cur-But (**B**). Mass spectra of BF_2_-cur-Ben (**C**) and BF_2_-cur-But (**D**) to AChE. (**E**) The possible responsive mechanism.

To explore the hydrolysis mechanism of AChE on the probes, we conducted HPLC and mass spectrometry (MS) experiments. Seen from Figure 3A,B, the retention time t_R_ is 6.1 min for BF_2_-cur-Ben, 4.8 min for BF_2_-cur-But, and 2.7 min for the hydrolysed product BF_2_-cur. After incubating BF_2_-cur-Ben with AChE, a new BF_2_-cur peak and benzoate ion (BI) peak could be observed, and the amount of BF_2_-cur and BI gradually increased with the increase of AChE concentration, while the amount of BF_2_-cur-Ben gradually decreased. Probe BF_2_-cur-But exhibited a similar trend between the probe and the product. As seen in Figure 3C,D, the peak found at *m*/*z* = 415.2908 [M − H]^−^ could correspond to the hydrolysed product BF_2_-cur, the peak found at 622.8762 [M − H]^−^ could correspond to the probe BF_2_-cur-Ben, and the peak found at *m*/*z* = 555.1364 [M − H]^−^ could correspond to the probe BF_2_-cur-But. Based on these, we proposed a responsive mechanism of the probes for AChE that the carboxylic ester bonds of BF_2_-cur-Ben and BF_2_-cur-But would be attacked by AChE, ultimately hydrolysing through the intermediate BF_2_-cur, shown in Figure 3E. 

**Figure 4:**
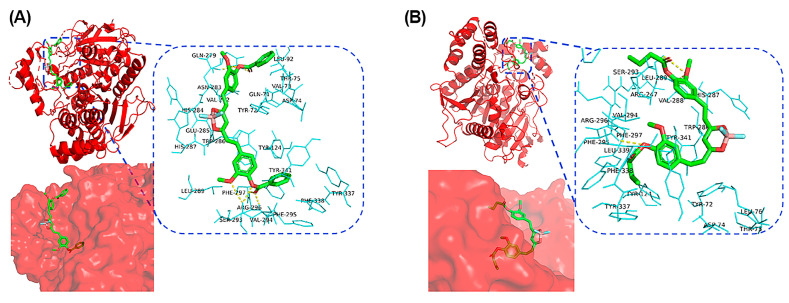
Binding model of (**A**) BF_2_-cur-Ben and (**B**) BF_2_-cur-But to AChE.

To better understand the recognition of the probes of AChE at the molecular level, we conducted molecular docking calculations. As shown in Figure 4A, most of the BF_2_-cur-Ben structure is embedded in AChE. The probe forms seven hydrogen bonds with the enzyme, which are distributed around the ester bonds on two sides. On one side there are three hydrogen bonds, which are formed with Ser-293 (two) and Arg-296 (one), and on the other side there are four hydrogen bonds with Asn-283 (two) and Gln-279 (two). For BF_2_-cur-But, as shown in Figure 4B, the probe is mostly embedded in AChE. BF_2_-cur-But forms only four hydrogen bonds with the enzyme, also distributed around the two-sided ester bonds. On one side, there is one hydrogen bond connected to Phe-295, and all three hydrogen bonds on the other side are formed with Leu-289. The binding free energy was calculated to be −8.9 kcal/mol for BF_2_-cur-Ben and −7.1 kcal/mol for BF_2_-cur-But, indicating that the two probes have good affinity with AChE. Obviously, BF_2_-cur-Ben can bind more stably to AChE because it possesses more hydrogen bonds and lower binding free energy, and this also theoretically verifies the results of the enzyme affinity test above (Enzyme kinetics section).

**Figure 5:**
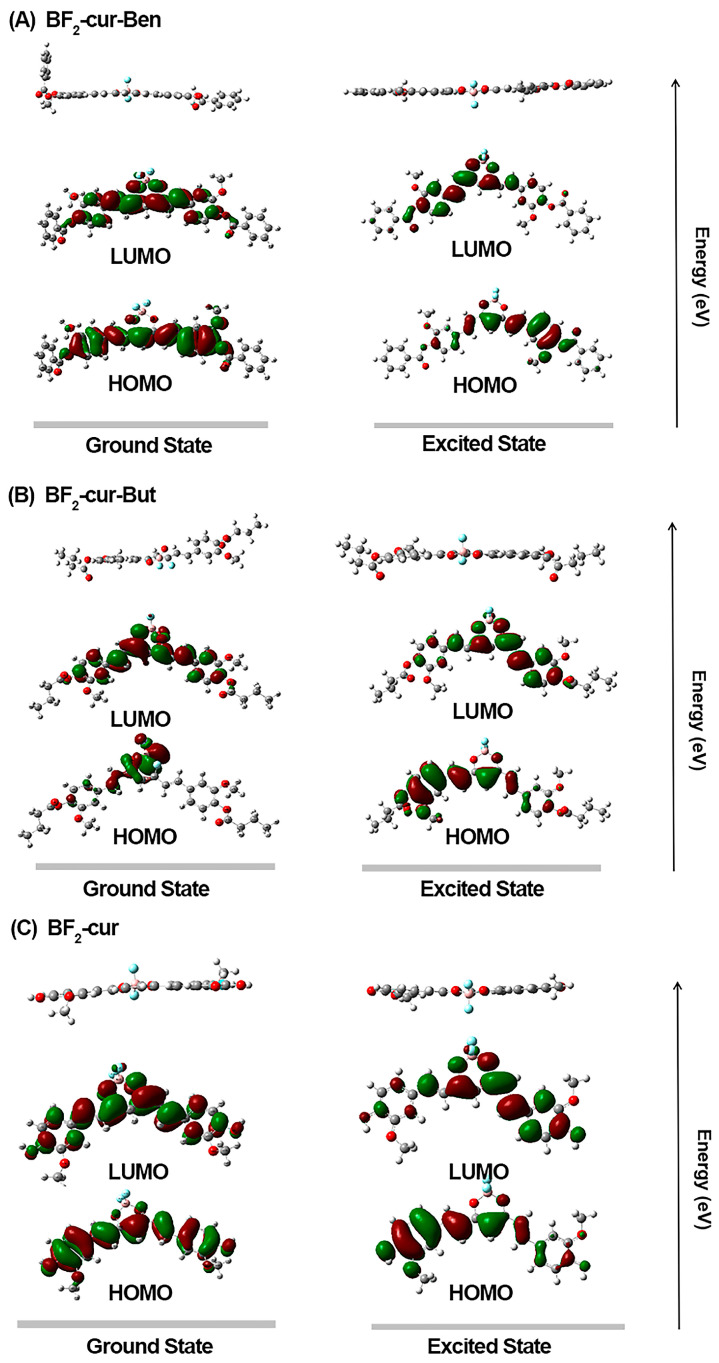
DFT and TD-DFT calculations of frontier molecular orbitals of (**A**) BF2-cur-Ben, (**B**) BF_2_-cur-But and (**C**) BF_2_-cur.

To further explain the fluorescence changes of the two probes before and after hydrolysis by AChE, we conducted DFT and TD-DFT calculations using Gaussian 16 software with the B3LYP/6-31G (d, p) set. As shown in Figure 5, the probes and product are typical D-A-D structures. Except for BF_2_-cur-But in the ground state, the electron density of HOMO is localized on the terminal phenyl rings possessing hydroxyl and methoxy groups. The higher electron density on the phenyl ring in HOMO explains their role as a donor core. The difluoroboron core possesses a higher electron density at LUMO and acts as an acceptor core. Therefore, the luminescence mechanism should belong to ICT, which is in agreement with the previously reported literature [43,44,45]. By comparing the optimized molecular structures under excited and ground states, for the two probes, the π-conjugated system within the molecule in the excited state is larger than the conjugated system in the ground state, which can undergo intramolecular charge transfer, and the electrons within the probe molecule can jump to the excited state and then back to the ground state, then can emit a strong fluorescence by itself, which is in agreement with other reports in the literature [42,46]. The conjugation system of the reacted product becomes smaller, and it becomes more difficult for the electrons to jump to the excited state, thus making it more difficult to emit light, thus producing the fluorescence quenching phenomenon.

### 2.3. Detection of AChE with the Probes at the Cellular Level

**Figure 6:**
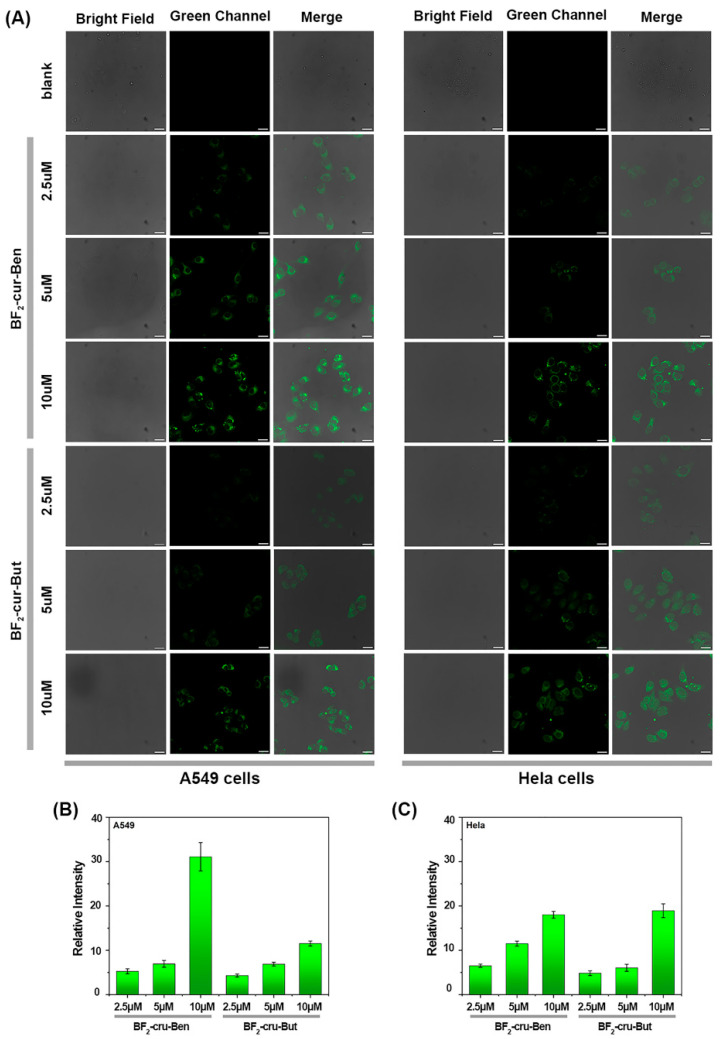
A549 and HeLa cells after incubation with different concentration of the two probes, respectively. (**A**) Confocal fluorescence images. (**B**,**C**) Quantification of the relative fluorescence intensities of the microscope images. Error bars are ± SD (*n* = 3), scale bar = 25 μm.

To explore whether the probes can penetrate the cell membrane and monitor intracellular AChE, cell imaging experiments were performed. As shown in Figure S6, a high survival rate (>80%) was maintained for multiple cell types (A549, HeLa, human hepatocellular carcinoma cells HepG2, human normal hepatocytes HL-7702, and mouse melanoma cells B16), which demonstrated that the probes have low biotoxicity and good biosafety, being suitable for application at the biological level. As shown in Figure 6, after adding the three different concentrations of probes to A549 and HeLa cells and incubating for 1 h, the cells were visibly “lit up” and gradually increased in brightness with the probe concentration, indicating that the two probes can gradually penetrate the cell membrane. The fluorescence of BF_2_-cur-Ben was more intense in both A549 and HeLa cells, indicating that this probe has higher cell membrane permeability. It is known that the membrane permeability of a compound is related to its oil-water partition coefficient (log *p* value). Generally, the larger the log *p* value, the more membrane permeable it is. The calculation shows that the log *p* value of BF_2_-cur-Ben (8.2422) > BF_2_-cur-But (6.3585), which is consistent with the report [47].

**Figure 7:**
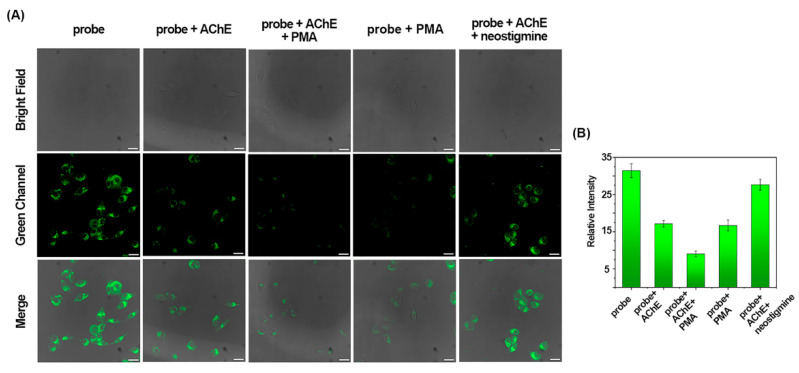
BF_2_-cur-Ben monitors accelerant/inhibitor-induced regulation of AChE in A549 cells. (**A**) Confocal fluorescence images. (**B**) Quantification of the relative fluorescence intensities of microscope images. Error bars are ± SD (*n* = 3), scale bar = 25 μm.

According to the reports, AChE is overexpressed during apoptosis, and the expressed AChE in turn continues to promote apoptosis [4,5,6,7,8,9,48]. AChE can enhance the sensitivity of non-small cell lung cancer cells to cisplatin-induced apoptosis and inhibit tumorigenesis of H520 cells in nude mice [49]. To date, AChE has emerged as a potential drug for lung cancer [50]. To this end, AChE activity in A549 cells was monitored by BF_2_-cur-Ben during apoptosis, where phorbol-12-myristate-13-acetate (PMA) was selected as an inducer to elicit apoptosis. As shown in Figure 7, when the promoter PMA was added to the cells, the intracellular fluorescence became significantly weaker than that in the control group. Neostigmine is a typical inhibitor of AChE, and when it is added to cells, the fluorescence quenched by AChE can be restored. This suggested that the intracellular fluorescence changes are indeed negatively correlated with the activity of AChE, and the intracellular AChE levels can be sensitively monitored by BF_2_-cur-Ben.

### 2.4. Detection of AChE with the Probes at the Living Organism Level

**Figure 8:**
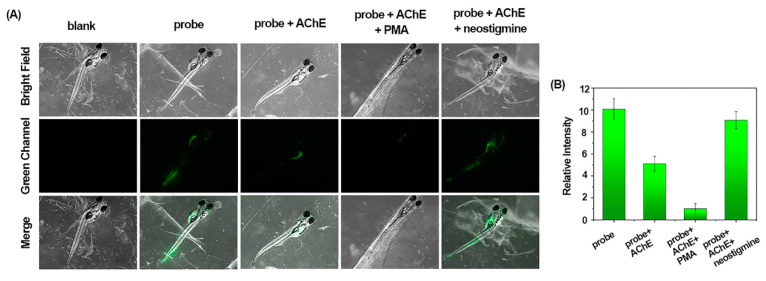
In vivo fluorescence imaging of BF_2_-cur-Ben for detection of AChE in zebrafish. (**A**) Confocal fluorescence images. (**B**) Quantification of relative fluorescence intensity of the microscopic images. Error bars are ± SD (*n* = 3).

Zebrafish are physiologically very similar to humans, they are easy to keep and mature quickly, and are a popular experimental model that enables direct observation. To further evaluate the potential of BF_2_-cur-Ben to visualize AChE in vivo, we carried out zebrafish experiments. As shown in Figure 8, after BF_2_-cur-Ben was incubated with zebrafish for 2 h, a green fluorescence signal was observed. The fluorescence in zebrafish pre-incubated with AChE significantly receded, and the fluorescence in zebrafish pre-incubated with AChE and then with PMA was weaker. However, when incubated with the inhibitor neostigmine after AChE pre-incubation, the fluorescence was significantly enhanced. These results suggest that BF_2_-cur-Ben can detect AChE at the living organism level and is a valuable visualization tool for monitoring the activity of AChE in organisms.

### 2.5. Comparison of Our Probes with Reported Sensors

Seen from Table 1, probes with fluorescent proteins and metal nanoclusters as luminescent moieties have not yet been able to be applied in the biological fields, although they can achieve a lower detection limit, while probes with organic fluorescent dyes as luminescent moieties can achieve the recognition of AChE in cells and animal tissues. The two probes in this paper can directly recognize AChE in vitro and in vivo without adding additional substrates or auxiliary substances, and their LOD is slightly lower.

## 3. Materials and Methods

### 3.1. Instruments and Reagents

A 600 MHz nuclear magnetic resonance instrument (Bruker, Saarbrucken, Germany), mass spectrometer (Thermo Fisher, Waltham, MA, USA), High-resolution mass spectroscope (Waters, Milford, MA, USA), ultraviolet spectrophotometer (Prodigy, Hudson, CT, USA), fluorescence spectrophotometer (Shimadzu, Kyoto, Japan), high-performance liquid chromatograph equipped with a UV/Visible detector (1260 LC, Agilent, Santa Clara, CA, USA), confocal fluorescence microscope (TCS SP5 II, Leica, Wetzlar, German), stereo fluorescence microscope (M205FA, Leica, Wetzlar, German) fully automated fluorescence enzyme labelling instrument (Infinite EPlex, Tecan, Mannedorf, Switzerland), and flow cytometer (Attune Nxt, Invitrogen, Carlsbad, CA, USA) were used. All reagents were purchased from legitimate commercial sources.

### 3.2. Synthesis

The synthetic route of BF_2_-cur-Ben and BF_2_-cur-But is shown in Scheme S1. Structural identification (^1^H NMR, ^13^C NMR and HRMS spectra) are shown in the Supplementary Information.

**Synthesis of BF_2_-cur** [51,52]. First, 1.473 g (4.0 mmol) of curcumin, 1.135 g (8.0 mmol) of boron trifluoride ether, and 1.841 g (8.0 mmol) of tributyl borate were accurately weighed and added to a round bottomed flask, and acetonitrile dried with anhydrous Na_2_SO_4_ was measured as the solvent to dissolve the compounds in a round bottomed flask. After nitrogen protection, the reaction system was stirred at reflux at 65 °C for 8 h. Subsequently, the solvent was evaporated under reduced pressure using a rotary evaporator to obtain the crude product solid. The crude product was purified by silica gel column chromatography and eluted with a solvent mixture of ethyl acetate:petroleum ether (5:3) to afford 1.420 g of dark red solid (i.e.,**BF_2_-cur**) in 85% yield.

^1^H NMR (500 MHz, DMSO-*d*_6_) δ 10.11 (s, 2H), 7.93 (d, *J* = 15.5 Hz, 2H), 7.48 (d, *J* = 2.0 Hz, 2H), 7.35 (dd, *J* = 8.3, 2.0 Hz, 2H), 7.02 (d, *J* = 15.6 Hz, 2H), 6.89 (d, *J* = 8.2 Hz, 2H), 6.46 (s, 1H), 3.86 (s, 6H). ^13^C NMR (151 MHz, DMSO) δ 179.17, 151.79, 148.63, 147.42, 126.44, 125.72, 118.31, 116.42, 112.83, 101.61, 56.21. MS (ESI) *m*/*z*, calculated value: 416.12, measured value: 416.4312.

**Synthesis of BF_2_-cur-Ben and BF_2_-cur-But.** First, 625 mg (1.5 mmol) of BF_2_-cur was accurately weighed and 40 mL of THF (dried over anhydrous Na_2_SO_4_) was measured as the solvent, and BF_2_-cur was completely dissolved in a round-bottomed flask. The round-bottomed flask was placed in an ice-water bath and 455 mg (4.5 mmol) of triethylamine was added dropwise. Then, 422 mg (3.0 mmol) benzoyl chloride/400 mg (3.75 mmol) butyryl chloride was dissolved in 10 mL of dichloromethane solution and added to the round-bottomed flask slowly with a dropping funnel over 30 min. Then, the ice bath was removed, and the reaction system was stirred at room temperature overnight, followed by evaporation of the solvent under reduced pressure using a rotary evaporator to obtain the crude product solid. The crude product was purified by silica gel column chromatography and eluted with a solvent mixture of dichloromethane: petroleum ether (8:1)/ethyl acetate: petroleum ether (5:1) to obtain 453.2 mg orange solid in 48% yield (**BF_2_-cur-Ben**)/544.8 mg dark red solid in 65% yield. (**BF_2_-cur-But**). **BF_2_-cur-Ben:** ^1^H NMR (500 MHz, Chloroform-*d*) δ 8.27–8.20 (m, 4H), 8.06 (d, *J* = 15.6 Hz, 2H), 7.71–7.65 (m, 2H), 7.55 (t, *J* = 7.8 Hz, 4H), 7.31 (dd, *J* = 8.2, 1.9 Hz, 2H), 7.28–7.21 (m, 4H), 6.73 (d, *J* = 15.6 Hz, 2H), 6.17 (s, 1H), 3.90 (s, 6H). ^13^C NMR (151 MHz, Chloroform-*d*) δ 164.38, 151.95, 146.92, 143.16, 133.81, 132.97, 132.54, 130.40, 128.96, 128.65, 123.85, 122.32, 120.72, 112.56, 102.33, 56.08. MS (ESI) *m*/*z*, calculated value: 624.18, measured value: 624.1660. **BF_2_-cur-But:** ^1^H NMR (600 MHz, DMSO-*d*_6_) δ 8.07 (d, *J* = 15.8 Hz, 2H), 7.67 (d, *J* = 1.9 Hz, 2H), 7.51 (dd, *J* = 8.2, 1.9 Hz, 2H), 7.29 (d, *J* = 15.7 Hz, 2H), 7.23 (d, *J* = 8.1 Hz, 2H), 6.65 (s, 1H), 3.86 (s, 6H), 2.57 (t, *J* = 7.3 Hz, 4H), 1.67 (q, *J* = 7.3 Hz, 4H), 0.99 (t, *J* = 7.4 Hz, 6H). ^13^C NMR (151 MHz, DMSO) δ 180.48, 171.28, 151.80, 146.80, 142.82, 133.48, 124.04, 123.32, 122.10, 113.72, 102.65, 56.53, 35.49, 18.46, 13.73. MS (ESI) *m*/*z*, calculated value: 556.21, measured value: 556.1970.

### 3.3. Spectroscopy Experiments

All UV and fluorescence measurements were performed in DMSO/PBS = 5/95 (*v*/*v*) buffer. The probe was dissolved in DMSO to obtain a 1 mM stock solution. Analytes were prepared in purified water, including AChE, BChE, CEs, trypsin, α-chymotrypsin, lysozyme, phospholipase, glucose oxidase, elastase: 1000 U/mL, BSA: 1 mg/mL, amino acid: 50 mM, anionic and cationic stock solution: 50 mM. All spectroscopic measurements were performed at 37 °C using a 5 μM probe unless otherwise stated. Fluorescence test conditions: excitation wavelength λ_ex_ = 476 nm, emission wavelength λ_em_ = 533 nm, slit width: ex/em = 10.0/3.0 nm (BF_2_-cur-Ben) and 1.5/10.0 nm (BF_2_-cur-But).

### 3.4. HPLC and MS Experiments on Samples after Incubation

An appropriate amount of probe (10 mg/mL) and AChE (1000 U/mL) were mixed with a solution (acetonitrile/PBS = 1/9, *v*/*v*) for a final probe concentration of 0.01 mg/mL. After incubation for some minutes in a 37 °C water bath, it was filtered by a 0.45 μm microporous filtration membrane and then injected directly into the sample. HPLC conditions: chromatographic column XDB-C18 (5 μm, 250 mm × 4.6 mm), mobile phase: methanol/water (containing 5% phosphoric acid) (85/15, *v*/*v*), column temperature 25 °C, flow rate of 1.0 mL/min, injection volume 10 μL, detector wavelength: 430 nm. MS experiments: probe (0.1 mM) was incubated with AChE (150 U/mL for BF_2_-cur-Ben and 500 U/mL for BF_2_-cur-But) in a 37 °C water bath, then washed three times with saturated NaCl solution, extracted with ethyl acetate, and then the organic phase was retained, dried by blowing with N_2_, and tested directly.

### 3.5. Theoretical Calculations and Analysis

The chemical structures, electron cloud distributions and electron-leap energy levels of the probe BF_2_-cur-Ben, BF_2_-cur-But and the final product BF_2_-cur were calculated using Gaussian 16 software based on density-functional theory (DFT) and time-dependent density functional theory (TD-DFT), respectively, by selecting the B3LYP/6-31g (d, p) basis group and generating the energy-optimized (lowest) conformations of the two by calculations in the ground state and excited state, respectively. The molecular docking software used was AutoDock 4.2.6. AChE and BChE protein structures were obtained from the PDB database (PDB: 4PQE and 1P0M). The receptor lattice dot box size chosen for docking was 70 dots × 64 dots × 68 dots with a lattice spacing of 0.375 Å. The probe conformation search process was performed using the Lamarckian Genetic Algorithm (LGA).

### 3.6. Cytotoxicity and Cell Imaging

Cells were provided by the Cell Bank of Shanghai Institute of Biochemistry and Cell Biology, Chinese Academy of Sciences. Cell viability was tested using 3-(4, 5-dimethylthiazole-2-yl)-5-(3-carboxymethyl ester)-2-(4-sulfophenyl) -2h-tetrazolium, endolium salts (MTS). Cancer cells (human non-small cell lung cancer cells A549 and human cervical cancer cells HeLa) were incubated with AChE (10 U/mL) for 2 h, PMA (1 μg/mL) or neostigmine (10 μM) for 30 min, probe (10 μM) for 1 h, and washed three times with PBS before imaging under a confocal microscope with the excitation wavelength as 488 nm. Each experiment consisted of at least three independent biological replicates, and each slice captured at least three images with different fields of view, and Image J was used to analyse the results.

### 3.7. Zebrafish Imaging

Zebrafish were incubated with AChE (25 U/mL) for 2 h, PMA (2 μg/mL) or neostigmine (10 μM) for 1 h, probe (10 μM) for 2 h, and washed three times with PBS and anesthetized with 0.03% tricaine before imaging under an inverted stereo fluorescence microscope with a 4-fold objective. Experimental repeatability is the same as above.

## 4. Conclusions

In this study, two “on–off” probes of AChE were designed and synthesized, in which the readily available curcumin derivative BF_2_-cur was selected as a fluorescent group and benzoyl ester and butyryl ester were used as recognition groups. Comparative studies in evaluation were carried out. The obtained probes exhibited good selectivity, pH independence in vitro in a range of 0.5~7 U/mL for BF_2_-cur-Ben and 0.5~25 U/mL for BF_2_-cur-But. Meanwhile, BF_2_-cur-Ben has a lower LOD (0.031 U/mL), higher enzyme affinity (K_m_ = 16 ± 1.6 μM) and higher inhibitor sensitivity. Response mechanisms of the probes toward AChE were proposed based on HPLC and MS experiments and calculations. In molecular simulation, BF_2_-cur-Ben formed seven hydrogen bonds and its binding free energy was −8.9 kcal/mol, while BF_2_-cur-But formed only four hydrogen bonds and its binding free energy was −7.1 kcal/mol. This result showed that BF_2_-cur-Ben would have a more stable enzyme affinity, which was mirrored by the results of the comparison of K_m_ values. These two probes were able to enter the cell membrane and successfully detect exogenous AChE. In comparison, BF_2_-cur-Ben exhibited better cell membrane permeability due to its greater log *p* value, which is in agreement with previous findings. The probe BF_2_-cur-Ben could successfully track AChE expression in PMA-induced apoptosis of lung cancer cells and its ability to monitor AChE in organisms was confirmed by detecting AChE activity in zebrafish. Since AChE has the potential to inhibit the proliferation of a wide range of cancer cells, this probe is expected to be a potential diagnostic and therapeutic tool for clinical diagnosis and pathological mechanistic studies of AChE-related diseases.

## Figures and Tables

**Table 1:** Detection ability and application of different fluorescent probes for AChE.

Luminophore	Linearity Range (U/mL)	LOD (U/mL)	Application	Ref.
Fluorescent protein	2.5 × 10^−5^–2 × 10^−3^	1.5 × 10^−5^	food	[17]
Carbon quantum dots	1.42 × 10^−2^–1.218 × 10^−1^	4.25 × 10^−3^	human serum, semen	[18]
Black phosphorus quantum dots	2 × 10^−4^–5 × 10^−3^	4 × 10^−5^	/	[19]
Silver nanoclusters	0–4 × 10^−3^	7.1 × 10^−5^	/	[20]
Ag^+^-modified Au nanoclusters	3 × 10^−5^–4 × 10^−3^	2.1 × 10^−5^	human serum	[21]
Pyrene (polymer PVS assist)	/	1.5 × 10^−2^	screening enzyme inhibitors	[22]
Benzothiazoles (Cu^2+^ and ATCh assist)	2.5 × 10^−5^–4 × 10^−3^	1.8 × 10^−5^	HEK293 cells	[24]
Hemicyanine (with ATCh as substrate)	0–1 × 10^−2^	2 × 10^−4^	HeLa cells, MCF-7 cells, mice	[25]
Hemicyanine	0–8.0	0.1173	PC12 cells, zebrafish	[26]
Boron dipyrrole fluoride	0–20	0.21	PC12 cells, brains of mice, mice	[27]
Tricyanofurans (TCF)	0–50	0.17	PC12 cells, zebrafish	[28]
Hemicyanine	0–20	0.36	PC12 cells, brains of mice	[29]
Hemicyanine	/	/	neuromuscular junctions	[31]
Phenoxazin	0–20	1.7 × 10^−2^	U87MG cells, brains of mice, mice	[32]
Curcumin difluoroboride	0.5–7	3.1 × 10^−2^	A549 cells, HeLa cells	This work
	0.5–25	8.7 × 10^−2^		

## Data Availability

The data underlying this article are available in the article and Supplementary Material.

## Supplementary Materials

The following supporting information can be downloaded at: https://www.mdpi.com/article/10.3390/molecules29091961/s1.

## References

[1] Sussman I.S.A.J. (2005). Acetylcholinesterase: ‘classical’ and ‘non-classical’ functions and pharmacology. Curr. Opin. Pharmacol..

[2] Sola I., Aso E., Frattini D., López-González I., Espargaró A., Sabaté R., Di Pietro O., Luque F.J., Clos M.V., Ferrer I. (2015). Novel Levetiracetam Derivatives That Are Effective against the Alzheimer-like Phenotype in Mice: Synthesis, in Vitro, ex Vivo, and in Vivo Efficacy Studies. J. Med. Chem..

[3] Oukoloff K., Chao S., CieslikiewiczBouet M., Mougeot R., Jean L., Renard P.Y. (2016). Improved Access to Huprine Derivatives Functionalized at Position 9. Eur. J. Org. Chem..

[4] Jiang H., Zhang X. (2008). Acetylcholinesterase and apoptosis A novel perspective for an old enzyme. FEBS J..

[5] Zhang B., Lu L., Zhang X., Ye W., Wu J., Xi Q., Zhang X. (2014). Hsa-miR-132 regulates apoptosis in non-small cell lung cancer independent of acetylcholinesterase. J. Mol. Neurosci..

[6] Ye W., Gong X., Xie J., Wu J., Zhang X., Ouyang Q., Zhao X., Shi Y., Zhang X. (2010). AChE deficiency or inhibition decreases apoptosis and p53 expression and protects renal function after ischemia/reperfusion. Apoptosis.

[7] Zhang X., Lu L., Liu S., Ye W., Wu J., Zhang X. (2013). Acetylcholinesterase deficiency decreases apoptosis in dopaminergic neurons in the neurotoxin model of Parkinson’s disease. Int. J. Biochem. Cell Biol..

[8] Gao W., Zhu H., Zhang J., Zhang X. (2009). Calcium signaling-induced Smad3 nuclear accumulation induces acetylcholinesterase transcription in apoptotic HeLa cells. Cell. Mol. Life Sci..

[9] Park S.E., Kim N.D., Yoo Y.H. (2004). Acetylcholinesterase plays a pivotal role in apoptosome formation. Cancer Res..

[10] Munoz-Delgado E., Montenegro M.F., Campoy F.J., Moral-Naranjo M.T., Cabezas-Herrera J., Kovacs G., Vidal C.J. (2010). Expression of cholinesterases in human kidney and its variation in renal cell carcinoma types. FEBS J..

[11] Zhao Y., Wang X., Wang T., Hu X., Hui X., Yan M., Gao Q., Chen T., Li J., Yao M. (2011). Acetylcholinesterase, a key prognostic predictor for hepatocellular carcinoma, suppresses cell growth and induces chemosensitization. Hepatology.

[12] Nieto-Cerón S., Vargas-López H., Pérez-Albacete M., Tovar-Zapata I., Martínez-Hernández P., Rodríguez-López J.N., Cabezas-Herrera J. (2010). Analysis of cholinesterases in human prostate and sperm. Chem. Biol. Interact..

[13] Li Z., Wang Y., Ni Y., Kokot S. (2014). Unmodified silver nanoparticles for rapid analysis of the organophosphorus pesticide, dipterex, often found in different waters. Sens. Actuators B-Chem..

[14] Andreani A., Burnelli S., Granaiola M., Guardigli M., Leoni A., Locatelli A., Morigi R., Rambaldi M., Rizzoli M., Varoli L. (2008). Chemiluminescent high-throughput microassay applied to imidazo[2,1-b]thiazole derivatives as potential acetylcholinesterase and butyrylcholinesterase inhibitors. Eur. J. Med. Chem..

[15] Singh A.P., Balayan S., Hooda V., Sarin R.K., Chauhan N. (2020). Nano-interface driven electrochemical sensor for pesticides detection based on the acetylcholinesterase enzyme inhibition. Int. J. Biol. Macromol..

[16] Li Y.P., Zhao R.X., Han G.Y., Xiao Y.M. (2018). Novel Acetylcholinesterase Biosensor for Detection of Paraoxon Based on Holey Graphene Oxide Modified Glass Carbon Electrode. Electroanalysis.

[17] Lei C., Wang Z., Nie Z., Deng H., Hu H., Huang Y., Yao S. (2015). Resurfaced Fluorescent Protein as a Sensing Platform for Label-Free Detection of Copper(II) Ion and Acetylcholinesterase Activity. Anal. Chem..

[18] Qian Z., Chai L., Tang C., Huang Y., Chen J., Feng H. (2016). A fluorometric assay for acetylcholinesterase activity and inhibitor screening with carbon quantum dots. Sens. Actuators B-Chem..

[19] Gu W., Yan Y., Pei X., Zhang C., Ding C., Xian Y. (2017). Fluorescent black phosphorus quantum dots as label-free sensing probes for evaluation of acetylcholinesterase activity. Sens. Actuators B-Chem..

[20] Li C., Wei C. (2017). DNA-functionlized silver nanoclusters as label-free fluorescent probe for the highly sensitive detection of biothiols and acetylcholinesterase activity. Sens. Actuators B-Chem..

[21] Wang M., Li N., Wang S., Chen J., Wang M., Liu L., Su X. (2021). Constructing self-assembled nanohybrids for the ratiometric fluorescent sensing of acetylcholinesterase activity. Sens. Actuators B-Chem..

[22] Chen J., Liao D., Wang Y., Zhou H., Li W., Yu C. (2013). Real-Time Fluorometric Assay for Acetylcholinesterase Activity and Inhibitor Screening through the Pyrene Probe Monomer–Excimer Transition. Org. Lett..

[23] Liao S., Han W., Ding H., Xie D., Tan H., Yang S., Wu Z., Shen G., Yu R. (2013). Modulated Dye Retention for the Signal-On Fluorometric Determination of Acetylcholinesterase Inhibitor. Anal. Chem..

[24] Zhang P., Fu C., Xiao Y., Zhang Q., Ding C. (2020). Copper(II) complex as a turn on fluorescent sensing platform for acetylcholinesterase activity with high sensitivity. Talanta.

[25] Zhao C., Zhou F., Lu K., Yang S., Tan B., Sun W., Shangguan L., Wang H., Liu Y. (2022). Near-infrared fluorescent probe for in vivo monitoring acetylcholinesterase activity. Sens. Actuators B-Chem..

[26] Ma J., Si T., Yan C., Li Y., Li Q., Lu X., Guo Y. (2020). Near-Infrared Fluorescence Probe for Evaluating Acetylcholinesterase Activity in PC12 Cells and In Situ Tracing AChE Distribution in Zebrafish. ACS Sens..

[27] He N., Yu L., Xu M., Huang Y., Wang X., Chen L., Yue S. (2021). Near-infrared fluorescent probe for evaluating the acetylcholinesterase effect in the aging process and dietary restriction via fluorescence imaging. J. Mat. Chem. B.

[28] Fortibui M.M., Jang M., Lee S., Ryoo I., Ahn J.S., Ko S., Kim J. (2022). Near-Infrared Fluorescence Probe for Specific Detection of Acetylcholinesterase and Imaging in Live Cells and Zebrafish. ACS Appl. Bio Mater..

[29] Wang X., Li P., Ding Q., Wu C., Zhang W., Tang B. (2019). Observation of Acetylcholinesterase in Stress-Induced Depression Phenotypes by Two-Photon Fluorescence Imaging in the Mouse Brain. J. Am. Chem. Soc..

[30] Masahiro Oe K.M.A.M. (2022). An activator-induced quencher-detachment_x0002_based turn-on probe with a cationic substratemoiety for acetylcholinesterase. Chem. Commun..

[31] Chao S., Krejci E., Bernard V., Leroy J., Jean L., Renard P.Y. (2016). A selective and sensitive near-infrared fluorescent probe for acetylcholinesterase imaging. Chem. Commun..

[32] Wu X., An J.M., Shang J., Huh E., Qi S., Lee E., Li H., Kim G., Ma H., Oh M.S. (2020). A molecular approach to rationally constructing specific fluorogenic substrates for the detection of acetylcholinesterase activity in live cells, mice brains and tissues. Chem. Sci..

[33] Lin L., Wang P., Zhao X.L. (2012). Study on Curcumin-induced Apoptosis in Ovarian Cancer Resistant Cell Lines COC1/DDP. J. Sichuan Univ..

[34] Wang C.C., Zhuang J., Feng F.B., Wei J.Y., Sun Y., LÜ Q.L., Han H.E., Sun C.G. (2015). Regulative research of Curcumin mediated angiogenesis mimicry of lung cancer cell by Wnt/β-catenin signaling pathway. Chin. J. Cancer Prev. Treat..

[35] Qin J.W., Chen L. (2015). Effect and Mechanism of Curcumin on Melanoma Growth and Angiogenesis. Chin. J. ETMF.

[36] Zhang S., Tang D., Zang W., Yin G., Dai J., Sun Y.U., Yang Z., Hoffman R.M., Guo X. (2017). Synergistic Inhibitory Effect of Traditional Chinese Medicine Astragaloside IV and Curcumin on Tumor Growth and Angiogenesis in an Orthotopic Nude-Mouse Model of Human Hepatocellular Carcinoma. Anticancer Res..

[37] Laali K.K., Rathman B.M., Bunge S.D., Qi X., Borosky G.L. (2016). Fluoro-curcuminoids and curcuminoid-BF2 adducts: Synthesis, X-ray structures, bioassay, and computational/docking study. J. Fluor. Chem..

[38] Laali K.K., Greves W.J., Correa-Smits S.J., Zwarycz A.T., Bunge S.D., Borosky G.L., Manna A., Paulus A., Chanan-Khan A. (2018). Novel fluorinated curcuminoids and their pyrazole and isoxazole derivatives: Synthesis, structural studies, Computational/Docking and in-vitro bioassay. J. Fluor. Chem..

[39] Laali K.K., Greves W.J., Zwarycz A.T., Correa S.S., Troendle F.J., Borosky G.L., Akhtar S., Manna A., Paulus A., Chanan-Khan A. (2018). Synthesis, Computational Docking Study, and Biological Evaluation of a Library of Heterocyclic Curcuminoids with Remarkable Antitumor Activity. Chem. Med. Chem..

[40] Abonia R., Laali K.K., Somu D.R., Bunge S.D., Wang E.C. (2020). A Flexible Strategy for Modular Synthesis of Curcuminoid-BF2/Curcuminoid Pairs and Their Comparative Antiproliferative Activity in Human Cancer Cell Lines. Chem. Med. Chem..

[41] Laali K.K., Zwarycz A.T., Bunge S.D., Borosky G.L., Nukaya M., Kennedy G.D. (2019). Deuterated Curcuminoids: Synthesis, Structures, Computational/Docking and Comparative Cell Viability Assays against Colorectal Cancer. Chem. Med. Chem..

[42] Tang X., Zhang Y., Wang Q., Li Z., Zhang C. (2024). Detection of acetylcholinesterase and butyrylcholinesterase in vitro and in vivo using a new fluorescent probe. Chem. Commun..

[43] Sherin D.R., Thomas S.G., Rajasekharan K.N. (2015). Mechanochemical synthesis of 2,2-difluoro-4,6-bis(β-styryl)-1,3,2-dioxaborines and their use in cyanide ion sensing. Heterocycl. Commun..

[44] Margar S.N., Rhyman L., Ramasam P., Sekar N. (2016). Fluorescent difluoroboron-curcumin analogs: An investigation of the electronic structures and photophysical properties. Spectrochim. Acta A.

[45] Canard G., Ponce-Vargas M., Jacquemin D., Guennic B.L., Felouat A., Rivoal M., Zaborova E., D’Aleo A., Fages F. (2017). Influence of the electron donor groups on the optical and electrochemical properties of borondifluoride complexes of curcuminoid derivatives: A joint theoretical and experimental study. RSC Adv..

[46] Zhao L., He X., Huang Y., Li J., Li Y., Tao S., Sun Y., Wang X., Ma P., Song D. (2019). A novel ESIPT-ICT-based near-infrared fluorescent probe with large stokesshift for the highly sensitive, specific, and non-invasive in vivo detection of cysteine. Sens. Actuators B-Chem..

[47] Lu J., Wang Q., Wang Z., Liu J., Guo Y., Pan C., Li X., Che J., Shi Z., Zhang S. (2022). Log P analyzation-based discovery of GSH activated biotin-tagged fluorescence probe for selective colorectal cancer imaging. Eur. J. Med. Chem..

[48] Zhang X.J., Yang L., Zhao Q., Caen J.P., He H.Y., Jin Q.H., Guo L.H., Alemany M., Zhang L.Y., Shi Y.F. (2002). Induction of acetylcholinesterase expression during apoptosis in various cell types. Cell Death Differ..

[49] Lu L., Zhang X., Zhang B., Wu J., Zhang X. (2013). Synaptic acetylcholinesterase targeted by microRNA-212 functions as a tumor suppressor in non-small cell lung cancer. Int. J. Biochem. Cell Biol..

[50] Yuan J.J., Shen Z.X., Mou N., Cao Y.Q. (2017). Use of Acetylcholinesterase, Butyrylcholinesterase or Their Mutants in the Preparation or Screening of Drugs for the Treatment of Tumors. China Patent.

[51] Kim E., Felouat A., Zaborova E., Ribierre J., Wu J.W., Senatore S., Matthews C., Lenne P., Baffert C., Karapetyan A. (2016). Borondifluoride complexes of hemicurcuminoids as bio-inspired push-pull dyes for bioimaging. Org. Biomol. Chem..

[52] Xie R.J., Liu X.G., Xu G.S., Liu Z.X. (2014). Synthesis of difluoroboron curcumin allylic ether and its application as fluorescent sensor for palladium(0). J. Zhejiang Univ..

