# Supplementary material for: Two Fluorescent Probes for Recognition of Acetylcholinesterase: Design, Synthesis, and Comparative Evaluation
Source: Molecules. 2024 Apr 25;29(9):1961. doi: 10.3390/molecules29091961 (PMC11085145; doi:10.3390/molecules29091961)
Supplement: Supplementary file 1 [file molecules-29-01961-s001.zip › molecules-2963897-supplementary.pdf]

Supporting Material

Two fluorescent probes for recognition of acetylcholinesterase: design, synthesis, and comparative evaluation

Xia Lin[1,2,3] Qingyuan Yi[1], Binyang Qing[4], Weisen Lan[1], Fangcheng Jiang[5], Zefeng Lai[5],Jijun Huang[6], Qing Liu[6], Jimin Jiang[6], Mian Wang[,4], Lianjia Zou[2], Xinbi Huang[2,] * and Jianyi Wang[1,3,]*

[1]Guangxi Key Laboratory of Special Biomedicine, Medical College, Guangxi University, Nanning, 530004, China
[2]Faculty of Pharmacy, Guangxi Health Science College, Nanning, 530023, China
[3]School of Chemistry and Chemical Engineering, Guangxi University, Nanning, 530004, China
[4]College of Life Science and Technology, Guangxi University, Nanning, 530004, China
[5]Pharmaceutical College, Guangxi Medical University, Nanning, 530021, China
[6]Guangxi Zhuang Autonomous Region Drug Administration, Nanning 530029, China

*Corresponding authors: huangxinbi333@163.com(X. Huang); jianyiwang@gxu.edu.cn(J. Wang)

1 **Synthetic route**

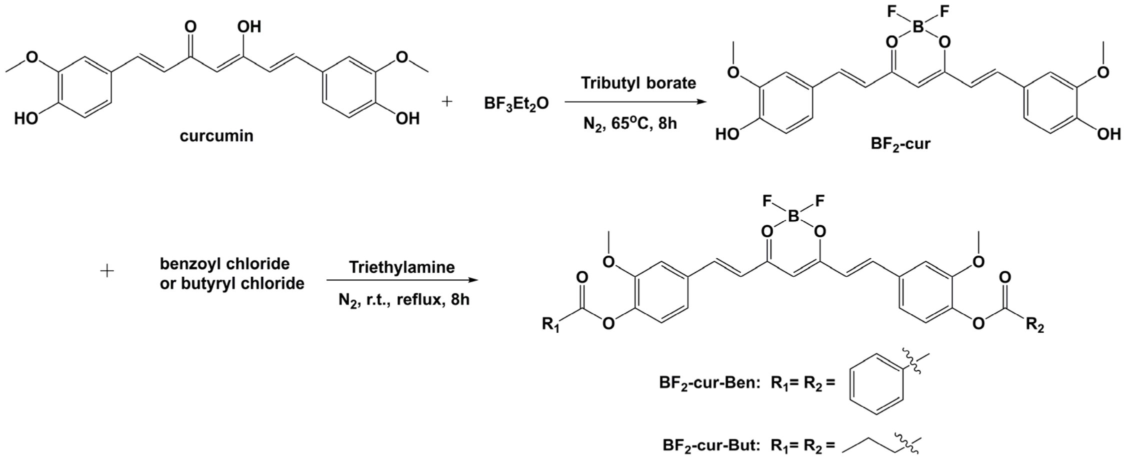

Scheme S1 Synthetic route of BF_2-cur-Ben and BF_2-cur-But.

2 **Response and detection of the probes on AChE *In vitro***

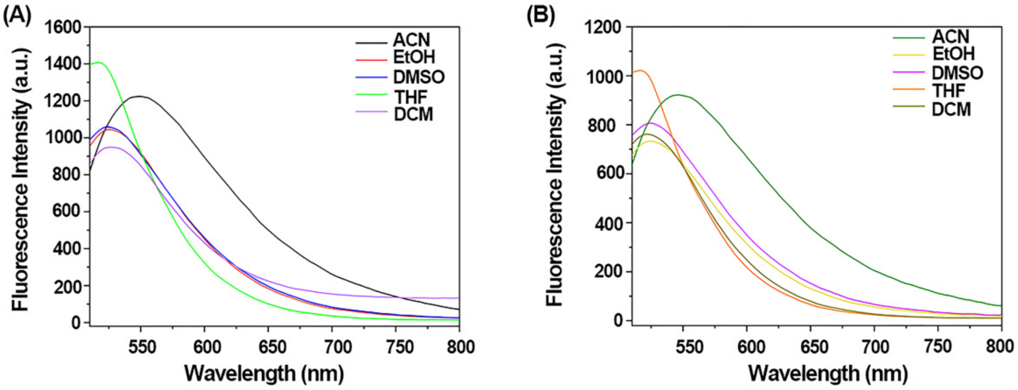

Figure S1. Fluorescence emission of BF_2-cur-Ben **(A)** andBF_2-cur-But **(B)** under different solvent conditions.

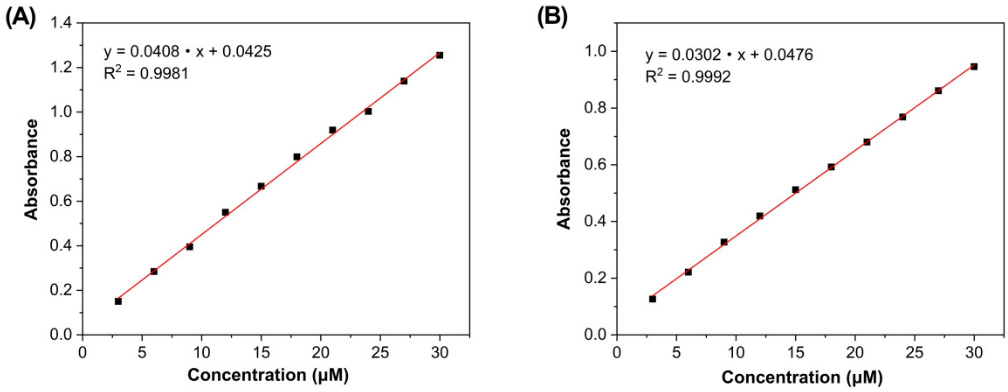

Figure S2. Lambert-Beer plot of BF_2-cur-Ben **(A)** and BF_2-cur-But **(B)**.

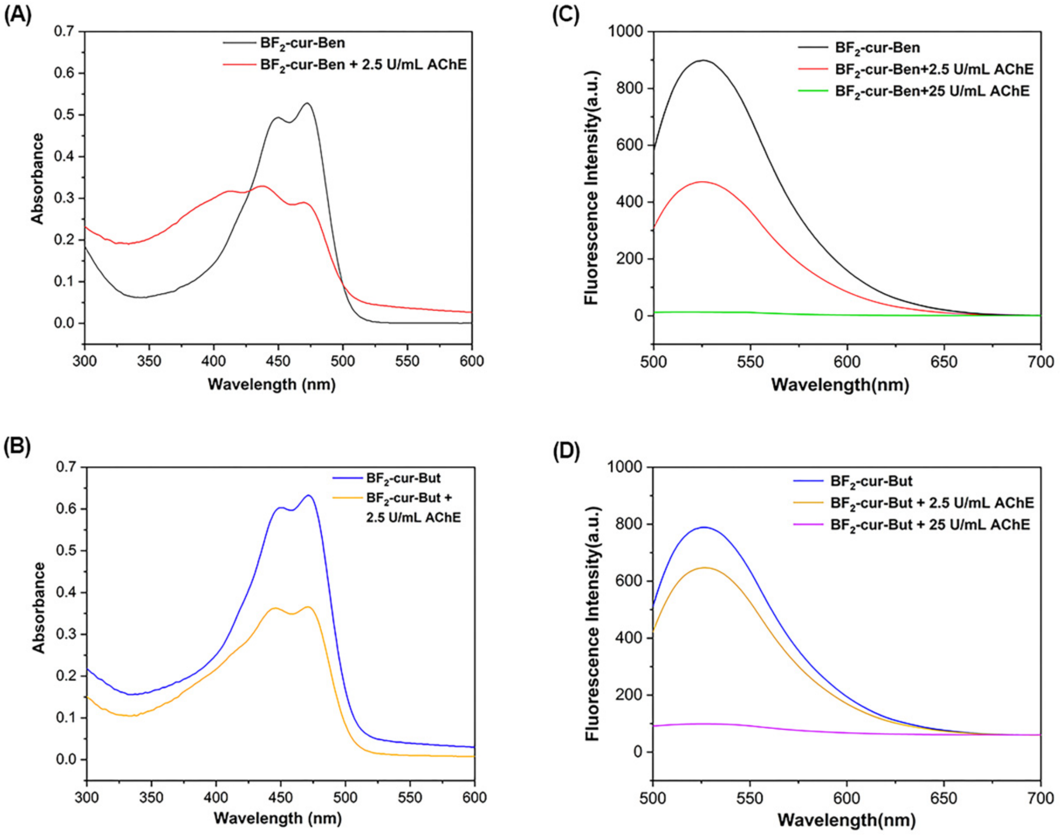

Figure S3. UV absorption **(A)(B)** and fluorescence spectra **(C)(D)** of BF_2-cur-Ben and BF_2-cur-But to AChE. The probes: 5 μM, AChE: 2.5 U/mL, Solvent: DMSO / PBS = 5 / 95 (V/V), pH = 7.4, incubation conditions: 37 °C, 60/20 min, λ_{ex} / λ_{em} = 476 nm / 533 nm, error bars are ± SD (n=3).

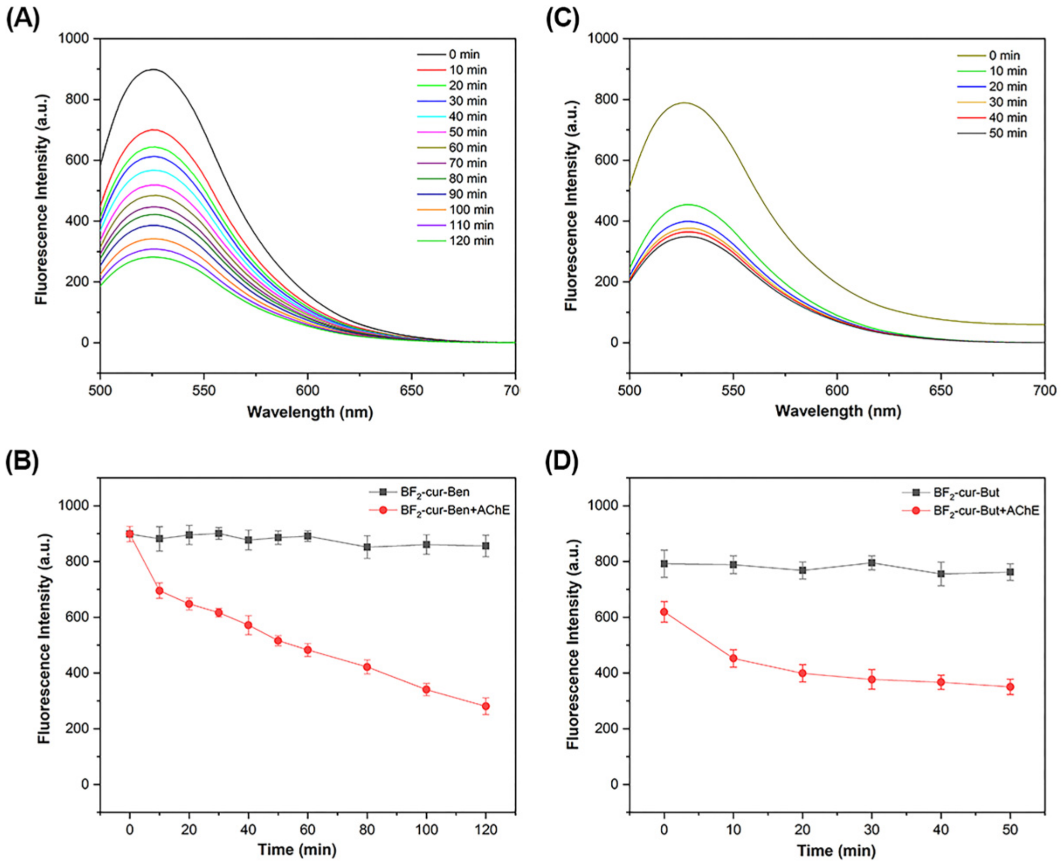

Figure S4. Fluorescence changes with incubation time for BF_2-cur-Ben **(A) (B)** and BF_2-cur-But **(C) (D)**. The probes: 5 μM, AChE: 2.5 U/mL, solvent: DMSO / PBS = 5 / 95 (V/V), pH = 7.4, incubation conditions: 37 °C, 0-120/50 min, λ_{ex} / λ_{em} = 476 nm / 533 nm, error bars are ± SD (n=3).

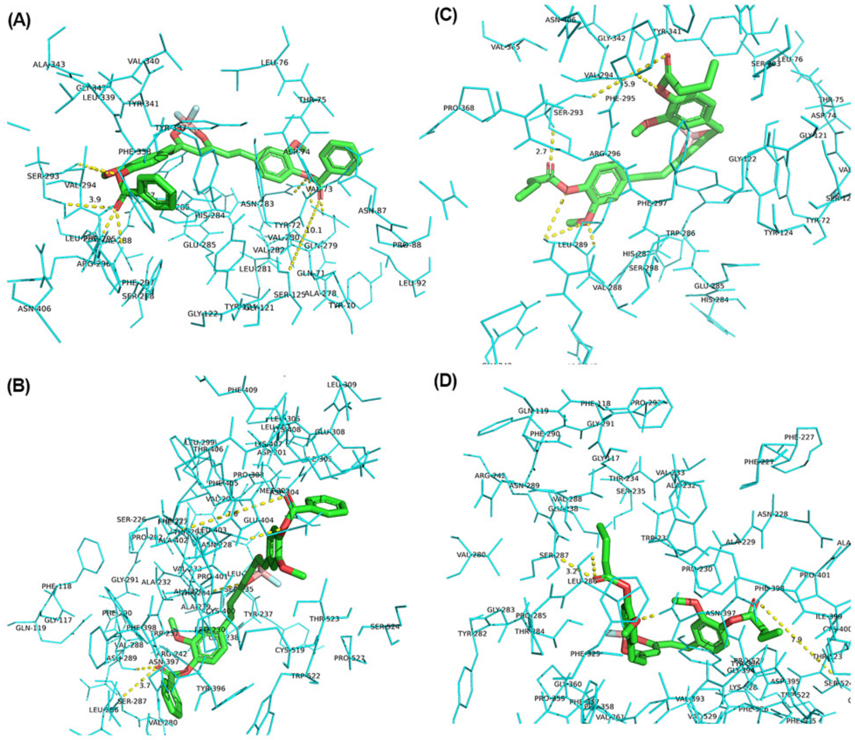

Figure S5. Molecular docking of BF$_2$-cur-Ben to AChE**(A)** and BChE**(B)**, and BF$_2$-cur-But to AChE**(C)** and BChE**(D)**.

Table S1 Results of molecular docking studies of the2 probes with AChE and BChE

		shortest distance for Ser pro-nuclear offense	hydrogen bonds
BF$_2$-cur-Ben	AChE	Ser-293:3.9 Å	7
	BChE	Ser-287:3.9 Å	3
BF$_2$-cur-But	AChE	Ser-293:2.7 Å	4
	BChE	Ser-287:3.2 Å	2

3 Safety Evaluation of the probes

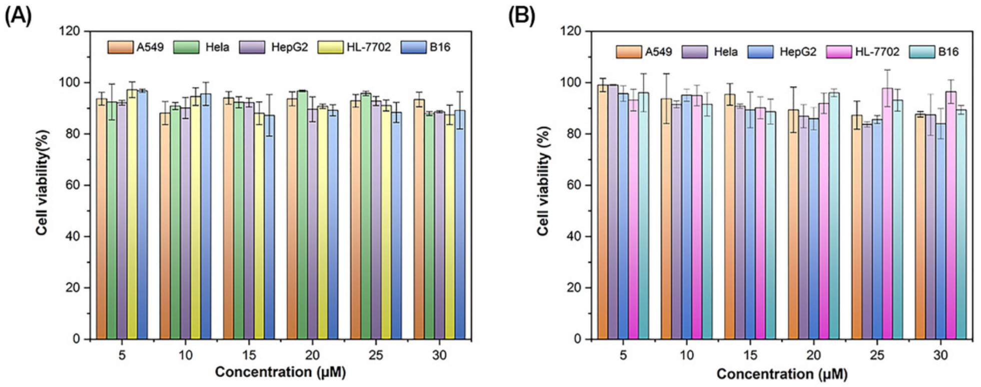

Figure S6. Cell survival of A549, Hela, HepG2, HL-7702 and B16 cells at differentprobe concentrations for BF_2-cur-Ben **(A)** andBF_2-cur-But **(B)**.

4 ^{1}H NMR spectra of the compounds

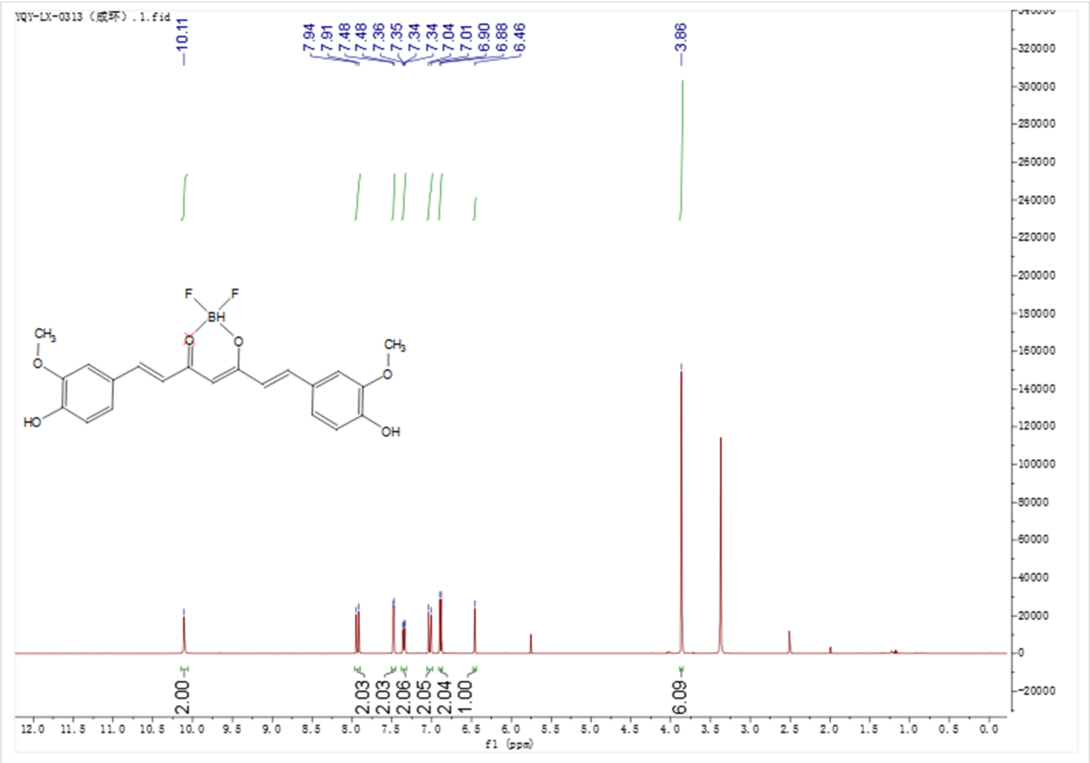

Figure S7. ^{1}H NMR spectrum of BF_2-cur in DMSO-d_6.

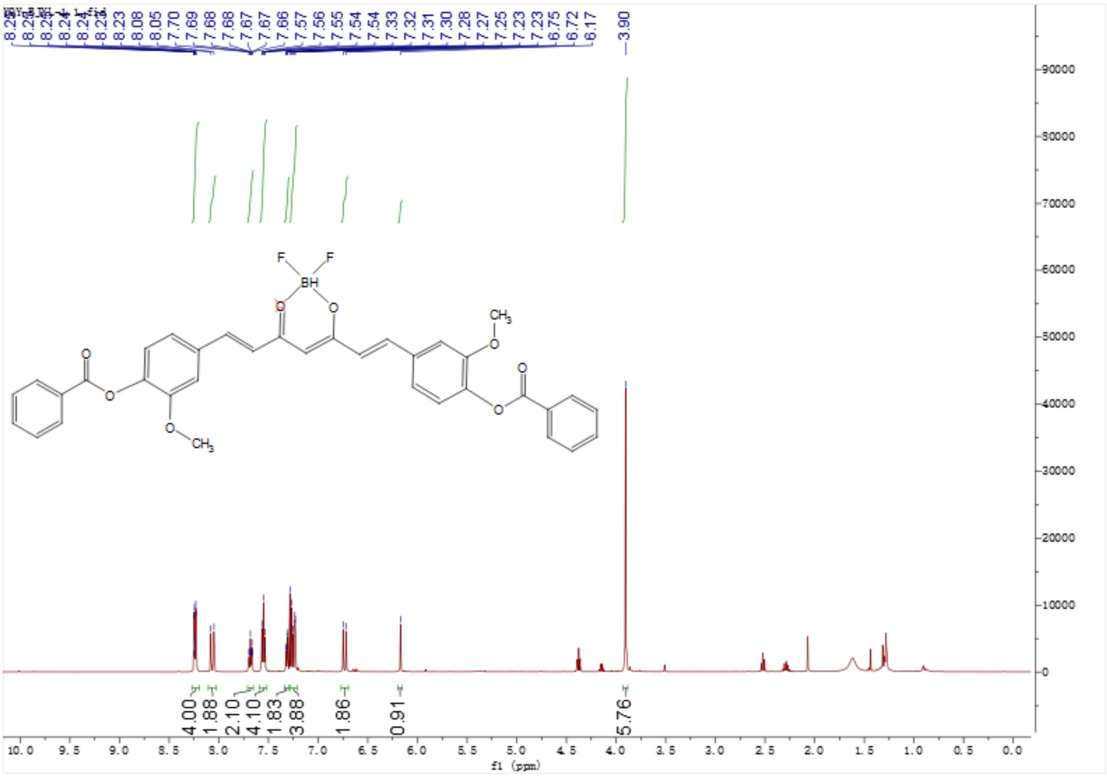

Figure S8. ^{1}H NMR spectrum of BF_2-cur-Ben in DMSO-d_6.

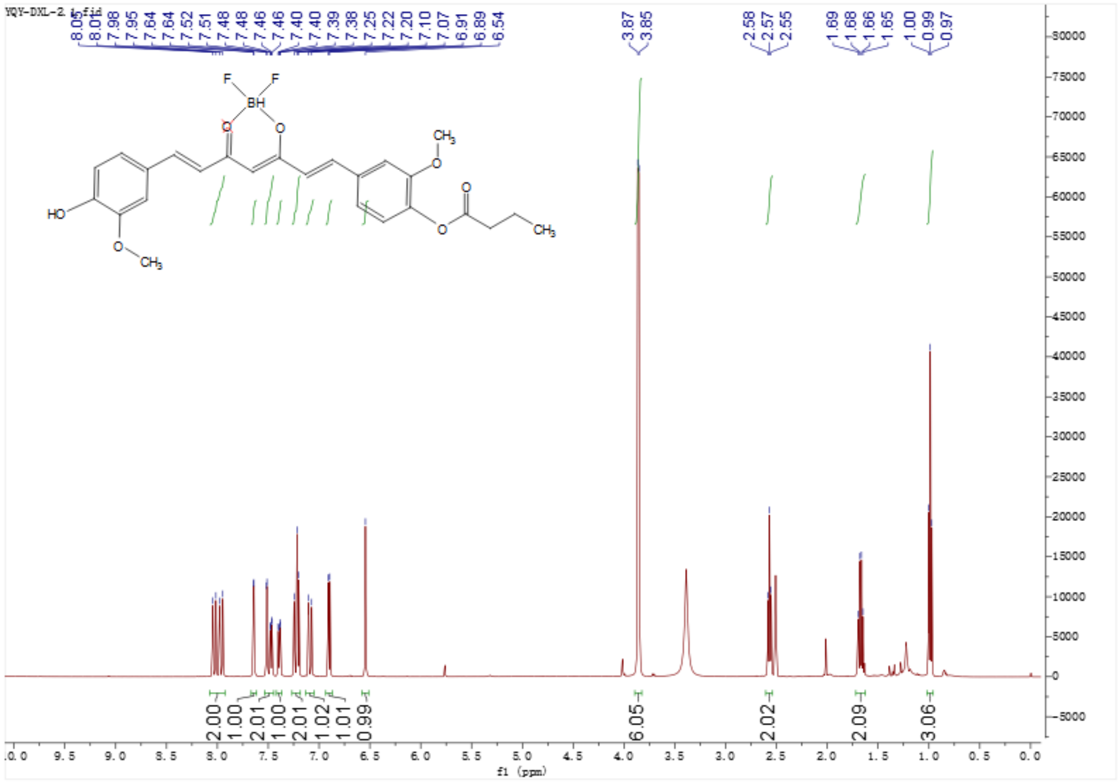

Figure S9. ^{1}H NMR spectrum of BF_2-cur-But in DMSO-d_6.

4 ^{13}C NMRspectra of the compounds

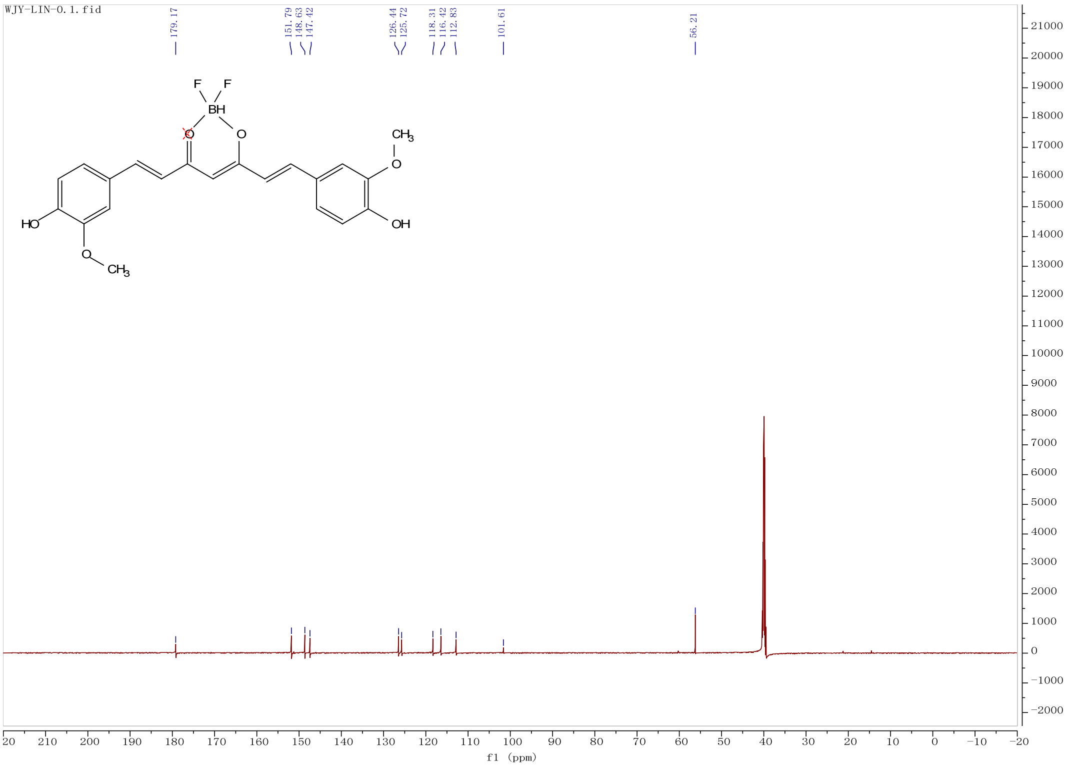

Figure S10. ^{13}C NMR spectrum of BF_2-cur in DMSO-d_6.

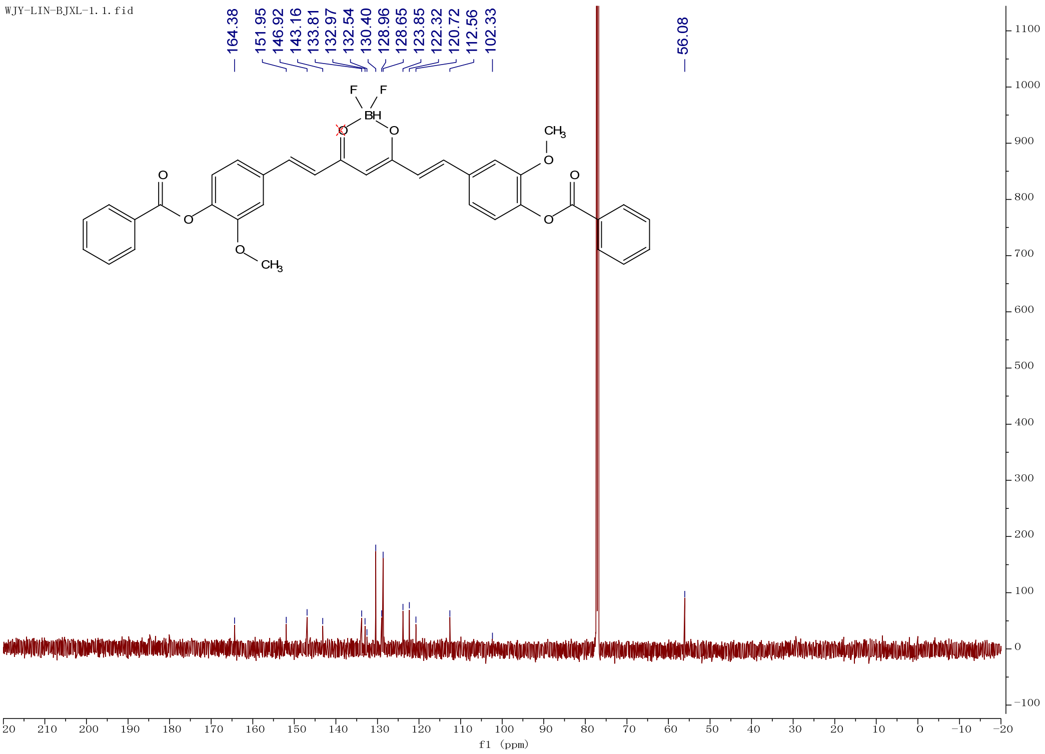

Figure S11. ^{13}C NMR spectrum of BF_2-cur-Ben in Chloroform-d.

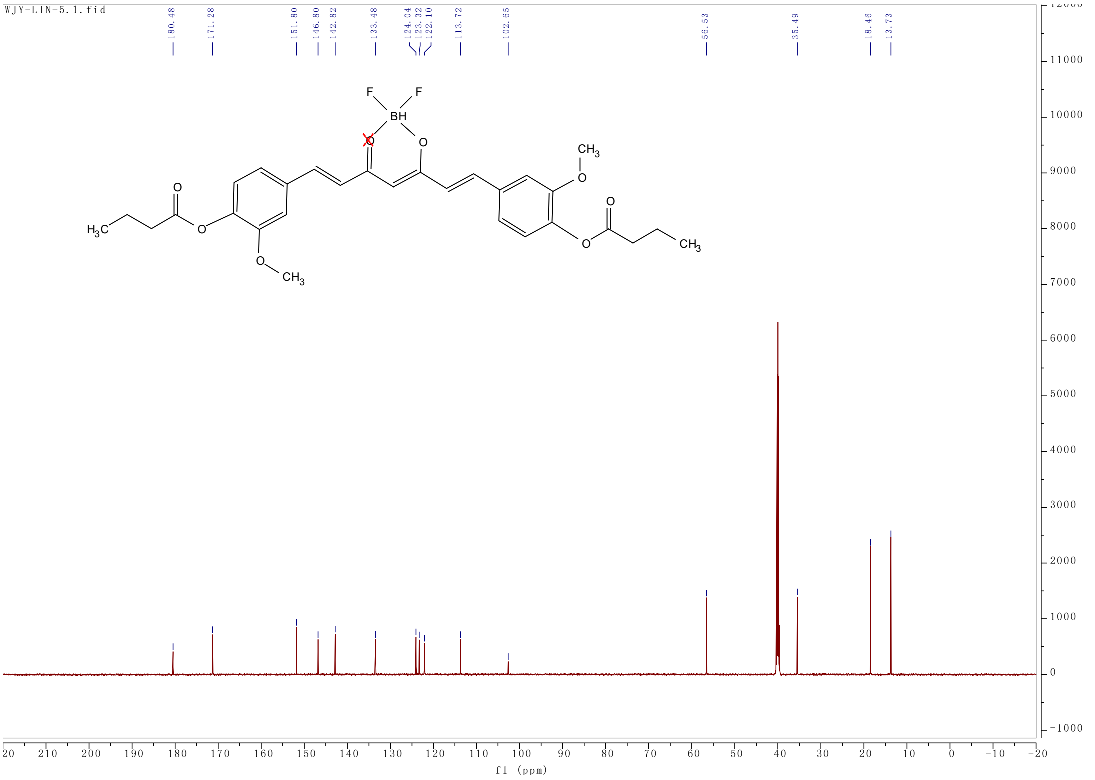

Figure S12. ^{13}C NMR spectrum of BF_2-cur-But in DMSO-d_6.

5 ESI-MS spectra of the compounds

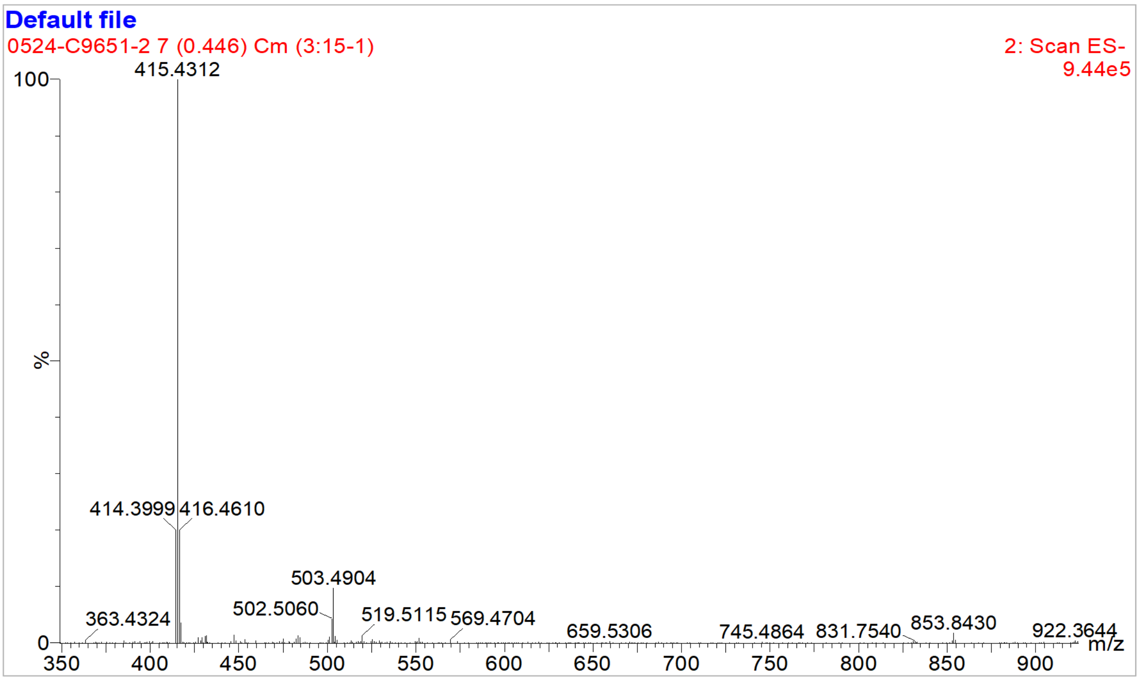

Figure S13. ESI-HRMS spectrum of BF_2-cur.

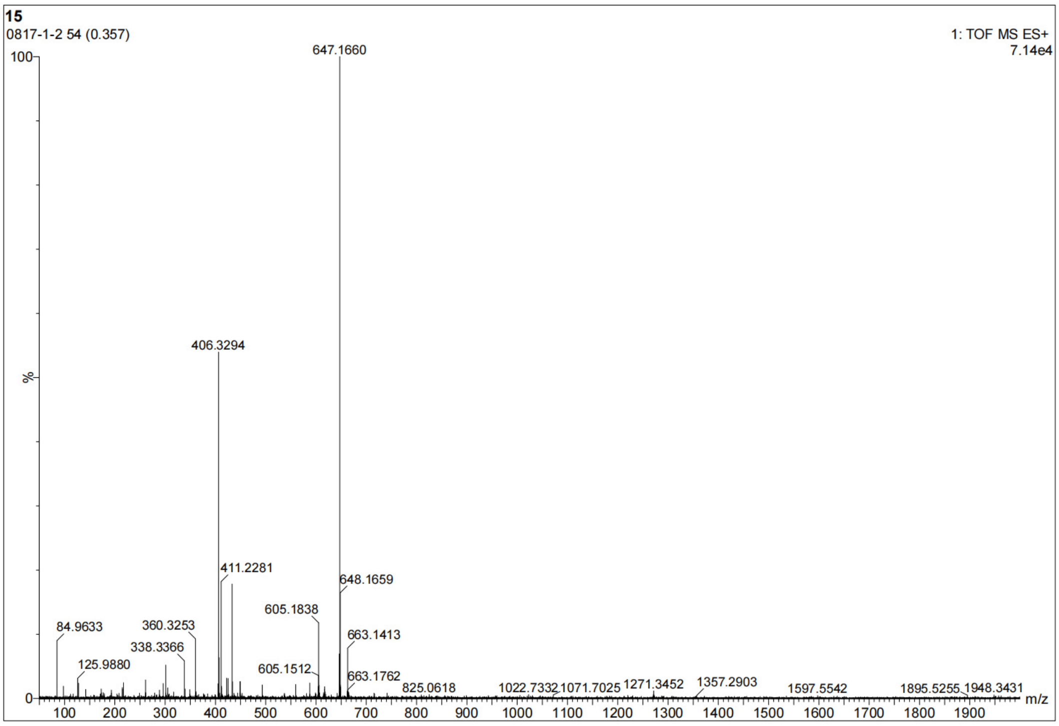

Figure S14. ESI-MS spectrum of BF_2-cur-Ben.

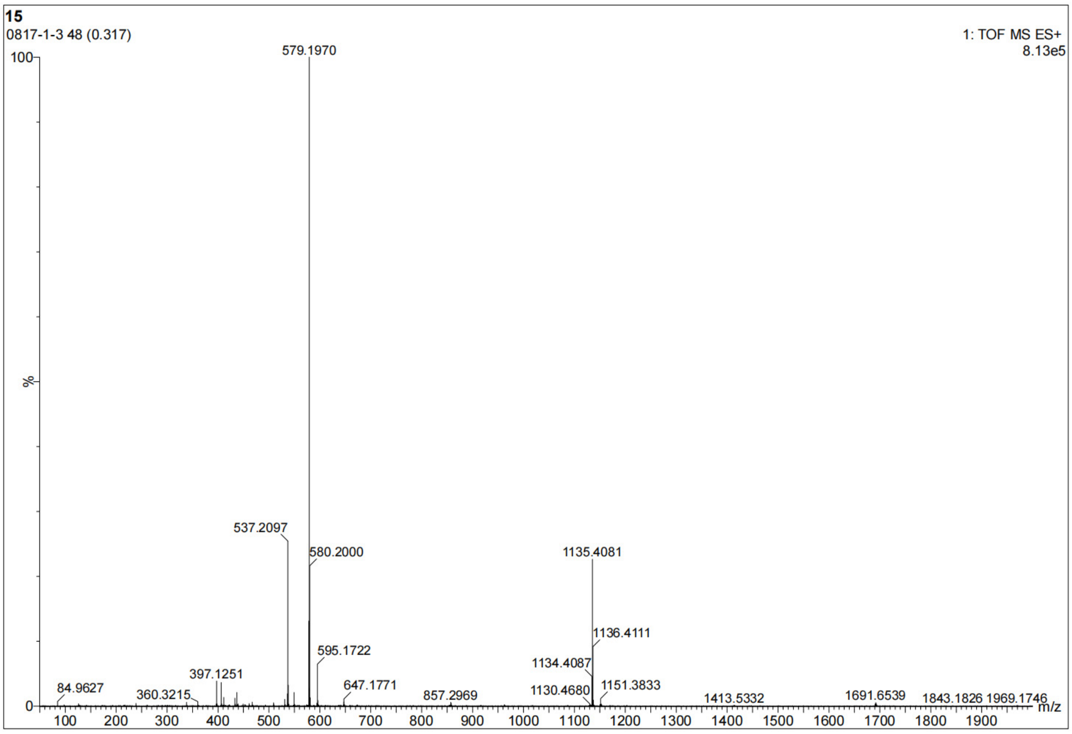

Figure S15. ESI-MS spectrum of BF_2-cur-But.
